# Efavirenz: New Hope in Cancer Therapy

**DOI:** 10.7759/cureus.67776

**Published:** 2024-08-25

**Authors:** Varshitha Dheep Elango, Uma Maheshwari Mugundan, Rajanandh MG

**Affiliations:** 1 Department of Pharmacy Practice, SRM (Sri Ramasamy Memorial) College of Pharmacy, SRM Institute of Science and Technology (Deemed to be University), Kattankulathur, IND

**Keywords:** novel anti-cancer, anti-retroviral, efavirenz, drug repurposing, breast cancer

## Abstract

Despite extensive research directed at preventive and treatment strategies, breast cancer remains the leading cause of cancer-related mortality among women. This necessitates the development of a new medication aimed at increasing patient survival and quality of life. A new drug's development from the ground up can cost billions of dollars and take up to ten or more years. Because much of the required safety and pharmacokinetic data are already available from earlier trials, repurposing medications usually results in lower costs and shorter turnaround times. Many antiretroviral medications target biological pathways and enzymes associated with cancer, which becomes an ideal option for repurposing as anticancer medications. Efavirenz is an antiretroviral medication that targets molecular pathways implicated in the growth of breast cancer, such as LINE-1 (long interspersed nuclear elements-1) suppression, hence lowering the proliferation of breast cancer cells and exhibiting anti-cancer properties. Additionally, it suppresses the fatty acid synthase gene and other important genes related to fat metabolism, impairing mitochondrial activity and making cancer cells deprived of energy. Efavirenz also inhibits cancer-initiating stem cells, promotes differentiation, and prevents recurrence. Additionally, efavirenz promotes oxidative damage by the formation of superoxide in cancer cells. In addition to its anti-cancer properties, efavirenz has the advantage of being a well-established and relatively inexpensive medication with a favorable safety profile. If proven effective, efavirenz could offer a cost-effective therapeutic option, which is an intriguing direction that warrants further investigation.

## Introduction and background

Breast cancer is defined by the World Health Organization (WHO) as an illness marked by an uncontrolled development of malignant breast cells. This indicates that aberrant cells emerge as a result of disruptions to the regular regulatory systems that govern cell growth and division. These cancerous cells have the ability to create tumors inside the breast tissue, which have the ability to metastasize, or spread, to other areas of the body. They may also infiltrate adjacent tissues [1]. As of 2022, breast cancer remains the most common cancer among women globally, with 2.3 million new cases diagnosed and 670,000 fatalities globally [2]. This underscores the need for continued research to develop new medications and treatments to improve survival rates, especially for advanced or metastatic stages of the disease. Breast cancer can develop resistance to existing treatments through various mechanisms, such as altering tumor microenvironment, epigenetic modifications, and increased drug efflux [3]. These resistance mechanisms reduce the effectiveness of current treatments, highlighting the need for research into new drugs and treatment approaches. Additionally, current treatments like chemotherapy and radiation have severe side effects, underscoring the importance of discovering novel therapies that can reduce these side effects and improve patients' overall quality of life.

Patients who are not responding to current treatments may find that research into novel medication classes offers them useful alternatives. Not every patient will benefit equally from the standard treatments for breast cancer, which include hormone therapy, radiation therapy, chemotherapy, and targeted therapies. This variation in the response to treatment highlights the necessity of ongoing innovation in the pharmaceutical industry. Investing in breast cancer research and developing new anticancer drugs is vital for tackling the challenges of this disease, offering hope for better treatment outcomes, increasing survival, and enhancing quality of life. It also contributes to the ultimate goal of curing breast cancer. Developing a new drug can take 10-15 years and billions of dollars, while repurposing existing drugs typically costs less and takes less time since much of the necessary safety and pharmacokinetic data is already available. Drug repurposing, sometimes referred to as drug repositioning, is the practice of treating cancer with drugs that were originally intended to treat other illnesses. By utilizing the current safety profiles and mechanisms of action of these medications, this approach may expedite the search for efficient cancer treatments.

Advances in molecular biology and genomics have shown that many biological pathways involved in various diseases are also implicated in cancer, and drugs targeting these pathways can be effective in cancer treatment. Observations from treating other diseases can provide valuable insights into the potential anticancer effects of these drugs. For example, the introduction of antiretroviral therapy in the mid-1990s significantly reduced cancer incidence among human immunodeficiency virus (HIV) patients. Since LINE-1 (long interspersed nuclear elements-1) retroviruses exhibit reverse transcriptase (RT) activity, antiretrovirals primarily used to treat HIV have shown promise in the treatment of cancer. There is a chance that some cancers will reactivate LINE-1 elements, which could cause genomic instability and accelerate the cancer. Non-nucleoside reverse transcriptase inhibitors (NNRTIs) decrease reverse transcription and the subsequent integration of LINE-1 segments into the genome by blocking LINE-1 reverse transcriptase. This can help mitigate the mutagenic effects of LINE-1 insertions, contributing to genomic stability. Downregulation of fatty acid metabolism was also found to contribute to their anticancer property.

Several antiretroviral drugs that were once created to treat HIV infection have demonstrated promise in focusing on the cellular processes and enzymes that contribute to the development of cancer. These medications provide promising new therapeutic options for cancer since they can affect a number of processes linked to the growth and survival of cancer cells. To sum up, antiretroviral drugs provide a new technique to treat cancer by influencing the immune system and focusing on vital pathways that are involved in DNA (deoxyribonucleic acid) synthesis and repair. Although there is a lot of promise, more investigation and clinical studies are necessary to completely grasp and capitalize on their advantages in cancer treatment.

Studies have demonstrated that antiretroviral medications, which were initially created to treat HIV, can also stop the development of different cancer cell lines. This strong evidence supports the need for more research into the potential of antiretroviral medications as cancer treatments. Research on cancer treatments has a potential direction because antiretroviral medications have been shown in preclinical trials to limit the proliferation of cancer cells and slow the progression of tumors. In order to confirm these effects in humans and possibly incorporate these medications into oncology practice, more research through clinical trials is necessary. These drugs could be combined with existing cancer therapies to prevent or overcome resistance, leading to more effective treatment regimens. While many antiretroviral drugs are being researched for cancer repurposing, this review focuses primarily on efavirenz.

## Review

Efavirenz anticancer mechanism by inhibiting long interspersed nuclear element-1

One of the most common autonomous retrotransposons, LINE-1 makes up about 17-21% of the human genome's sequence. Repeated DNA sequences known as LINE-1 elements are able to replicate and insert themselves into different parts of the genome. The stability and function of the genome are significantly impacted by their existence and activity [4]. Most breast carcinoma cell and biopsy specimens excessively express LINE-1, which is closely associated with the formation of various human malignancies [5]. Even though the processes behind LINE-1's expression in many tumors are still unclear, this protein is regarded to be a possible marker for many epithelium cancers. One of the potentially effective treatment approaches for breast cancer is expected to inhibit LINE-1 by repurposing antiretroviral medications as chemotherapeutic agents. Inhibiting this LINE-1 can inhibit dedifferentiation in several progenitors and transformed cell types [6]. Moreover, LINE-1 suppression has been connected to the regulation of a number of short non-coding RNA, which may affect not only the expression in oncogenes but also the synthesis of proteins linked with the growth of malignancy. Approximately 21% of the human genome is made up of LINE-1 retrotransposons, the most common source of RT activity. Therefore, RT inhibitors such as nucleoside/nucleotide reverse transcriptase inhibitors (NRTIs) and non-nucleoside/nucleotide reverse transcriptase inhibitors (NNRTIs) are thought to primarily target LINE-1 encoded RT.

The NNRTIs such as nevirapine and efavirenz interfere with LINE-1 reverse transcription, which has been well studied. Recent research indicates that efavirenz's higher affinity for LINE-1 encoded RT makes it a more powerful blocker of RT function. Reports state that efavirenz inhibits the function of the LINE-1 encoded RT protein and promotes differentiation in cancer A-375 cells, similar to the effects of short interfering RNA targeting LINE-1 elements [7]. Furthermore, efavirenz-inhibited LINE-1 action lowers the levels of recognized cancer genes in breast cancer, such as EGFR (epidermal growth factor receptor) and ERBB4 (erb-B2 receptor tyrosine kinase 4). In the T47D (tumor 47 ductal carcinoma) breast cancer cell line, silencing LINE-1 messenger RNA with a particular endo-small interfering RNA (endo453) raises a subset of tumor suppressor micro RNAs. Such micro RNAs are known for their ability to down-regulate a range of cancers and are linked to cell differentiation, Inhibition of metastasis, and prevention of carcinogenesis [8,9]. As a result, LINE-1 may be an unusual but interesting target for breast cancer treatment, as it is probably essential for the regulation of cancer cells. Reverse transcriptase activity expressed by LINE-1 has been shown to have major impacts on tumor cells, including growth suppression, differentiation, and morphological changes, according to research utilizing cell lines. It has been shown that efavirenz can reprogram several genes' transcription, resulting in the stimulation of gene expression related to differentiation and the restoration of differentiation and developmental processes in breast cancer cells. In conclusion, efavirenz has shown that it is capable of reprogramming gene expression in breast cancer cells, resulting in the production of gene profiles appropriate to differentiation and the restoration of typical developmental processes. These mechanisms hold promise for efavirenz as a potential treatment drug for breast cancer. Additionally, studies showed that the absence of efavirenz caused cell division to nearly revert to normal levels, suggesting that the drug's effects on cell growth are reversible. It has been demonstrated that efavirenz therapy reduces the proliferation of T47D as well as MCF (Michigan Cancer Foundation) 7 tumor cell lines via blocking LINE-1 [10,11]. By this LINE-1 inhibition, efavirenz exerts its anticancer property**. **

Efavirenz anticancer mechanism by inhibiting cancer stem cells

Cancer stem cells (CSCs) are essential for the growth of tumors, the return of cancer, and the development of resistance to radiation and chemotherapy. Their ability to change phenotype and function makes them difficult to eradicate. Eliminating CSCs is considered vital for effective cancer treatments due to their roles in metastasis, drug resistance, and recurrence. Thus this study shows that, with efavirenz, it is possible to not only eliminate primary breast cancer cells but also to promote changes in cell morphology. Research on CSCs has shown that efavirenz treatment significantly decreases the amount and size of tumorspheres and lowers the quantity of CSCs of the epithelial type. A number of genes associated with CSCs are upregulated by efavirenz, such as the CSC suppressor gene micro RNA-182 and the CSC marker micro RNA-21. Efavirenz administration decreased the number of tumorspheres by almost 20 times in MDA-MB-231 (MD Anderson-Metastatic Breast-231 cells), 15 times in T47D cells, 11 times in MCF10CA1α cells, and eight times in MCF10AT cells in experiments involving different cell lines. These findings indicate that efavirenz can significantly diminish functional CSCs, impacting both CSCs and non-CSCs. In summary, efavirenz exhibits significant therapeutic potential through its impact on the phenotypic and gene expression of CSCs, in breast cancer stem cells. This antiviral drug has the ability to stimulate the differentiation of breast CSCs in addition to aiding in the elimination of the initial breast cancer cells. In conclusion, by encouraging the differentiation of breast CSCs in addition to targeting and eliminating initial breast cancer cells, this antiretroviral drug efavirenz presents a potentially effective treatment approach that could enhance the overall management of breast cancer [12].

Efavirenz anticancer mechanism by fatty acid metabolism pathway

Efavirenz was thoroughly tested in triple-negative breast cancer (TNBC) cell lines to assess its impact on various cellular properties. By reviewing several crucial variables, including cellular characteristics, morphological changes, and LINE-1 expression, the study sought to comprehend how efavirenz affects TNBC cells. In conclusion, a thorough comprehension of efavirenz's effects and possible therapeutic efficacy can be obtained by analyzing its evaluation in TNBC cell lines through measurements of morphological alterations, survival, proliferation, mortality, and LINE-1 expression. These investigations are essential for figuring out potential uses of efavirenz in the treatment of TNBC and for figuring out any underlying mechanisms of action.

Efavirenz induces apoptosis, inhibits cellular growth, and modifies the morphology of the cells to mimic epithelial cells in certain cell lines. Using whole-genome RNA sequencing, it was discovered that the lipid metabolic route is an essential controller of efavirenz's anticancer activities [13]. This pathway's downregulation, which is closely linked to the emergence of cancer, is important to the way that efavirenz works. The downregulation of fatty acid metabolism is intimately associated with efavirenz's anticancer actions in TNBCs. RNA-sequence data across multiple TNBC cell lines demonstrated that the drug markedly downregulated several key genes related to fatty acid metabolisms, such as stearoyl-CoA desaturase (SCD), acyl-CoA synthetase long-chain family member 5 (ACSL5), fatty acid synthase (FASN), acyl-CoA synthetase long-chain family member 3 (ACSL3), fatty acid desaturase 1 (FADS1), protein tyrosine phosphatase like B (PTPLB), and acetyl-CoA carboxylase alpha (ACACA). Acetyl-CoA carboxylase (ACC), an enzyme necessary for the first committed step of fatty acid synthesis, is inhibited by efavirenz. Acetyl-CoA is changed by ACC into malonyl-CoA, an initial step in the production of fatty acids. Inhibiting ACC reduces malonyl-CoA availability, thus limiting fatty acid synthesis [14].

Inhibition of fatty acid metabolism by efavirenz contributes to mitochondrial dysfunction, exacerbating the energy crisis in cancer cells. Efavirenz disrupts mitochondrial beta-oxidation of fatty acids, a key adenosine triphosphate (ATP)-generating process, thereby reducing the energy supply available to cancer cells. This energy deprivation hampers cancer cell proliferation and survival. Inhibition of fatty acid production disturbs lipid biosynthesis, which affects membrane integrity and cell signaling. Cancer cells require lipids for the creation of their cell membranes and other essential components. Because of its capacity to alter the phenotype of CSCs, it triggers differentiation and specifically targets primary cancer cells, efavirenz is a prospective option for additional investigation in breast cancer therapy. Further research into its therapeutic potential may open the door to more innovative and potent therapy choices for those with breast cancer. The disruption of fatty acid metabolism induces metabolic stress in cancer cells, potentially activating apoptotic pathways, particularly the intrinsic (mitochondria-mediated) pathway. Inhibition of fatty acid metabolism can increase reactive oxygen species (ROS) production, contributing to oxidative stress and promoting apoptosis in cancer cells. Efavirenz’s anticancer properties through inhibition of fatty acid metabolism involve targeting key enzymes like ACC and FASN, disrupting mitochondrial function, and causing energy deprivation. This leads to metabolic stress, apoptosis induction, inhibition of cell proliferation, and targeting of cancer stem cells. These mechanisms make efavirenz a promising candidate for repurposing as an anticancer agent, especially in combination with other therapies to enhance treatment efficacy [15].

Efavirenz anticancer mechanism by mitochondrial damage

**Figure 1:**
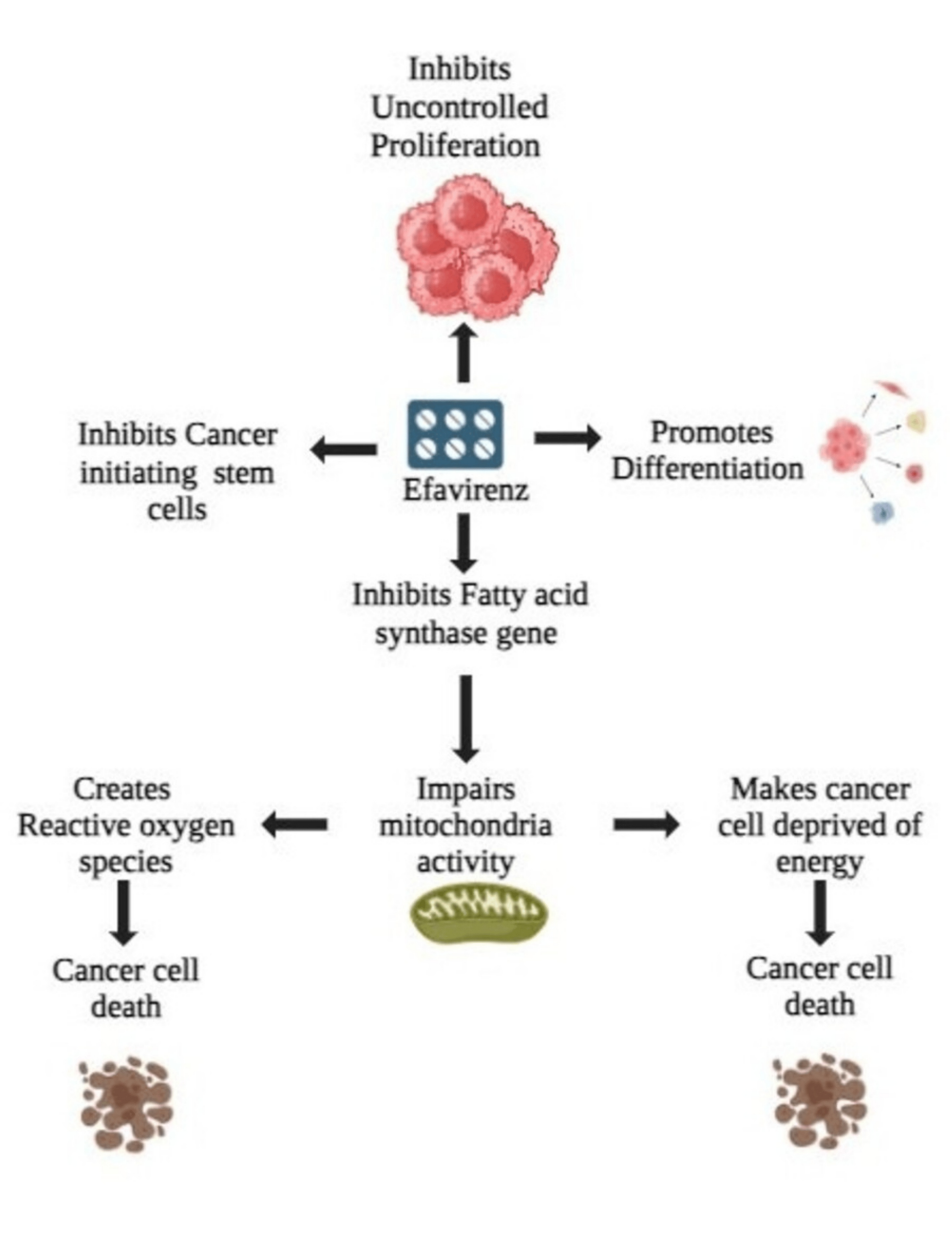
Anticancer mechanism of efavirenz Image Credits: Varshitha Dheep Elango, Uma Maheshwari Mugundan

Efavirenz inhibits the pathway of electron transport complex 1 and mitochondrial membrane potential, which increases the production of superoxide in cells and results in oxidative damage. It has reversible effects on mitochondrial activity, lowering the potential of the mitochondrial membrane and raising the formation of superoxide inside the mitochondria, which is followed by a decrease in the glutathione content within the cell. Antioxidant pretreatment can partially counteract this harmful effect, suggesting that ROS production plays a role in efavirenz's effects. Efavirenz's free radical properties suggest its potential as an anticancer drug, particularly in combination with radiotherapy due to its radio-sensitizing effects. Enzymes in the mitochondria electron transfer chain, specifically complexes I and II, are inhibited, which reduces mitochondrial ATP synthesis and REDOX (reduction-oxidation) activity [16]. The usual functioning of the electron transport chain is disrupted by this inhibition, which results in a decrease in ATP generation and a rise in the production of ROS. Efavirenz may also interfere with mitochondrial DNA polymerase gamma (POLG), impairing mitochondrial DNA replication and function. When the electron transport pathway is disrupted, ROS builds up and causes reactive destruction of proteins, DNA, and lipids in the mitochondria along with other parts of cells [17]. Efavirenz causes depolarization of the mitochondrial membrane potential, which is critical for maintaining mitochondrial function and integrity. This disruption can result in mitochondrial dysfunction and cell death. The intrinsic mitochondria-mediated apoptotic pathway activates caspases and results in programmed cell death, which can be triggered by elevated ROS generation. Through this pathway, pro-apoptotic molecules like cytochrome C are released from mitochondria and enter the cytosol [18,19]. These are the various mechanisms by which efavirenz exhibits its anticancer properties, which are depicted in Figure 1. 

## Conclusions

Although preclinical evidence indicates that efavirenz, an antiviral medication mostly used to treat HIV, may have anti-cancer properties, more investigation and clinical studies are necessary to adequately assess efavirenz's potential as a cancer treatment. While efavirenz has demonstrated promise in a number of preclinical studies, suggesting potential mechanisms by which it may impact cancer cells, a number of critical stages need to be completed before efavirenz can be regarded as a feasible therapeutic option for breast cancer. To sum up, although preclinical evidence suggesting that efavirenz has anti-cancer properties is encouraging, comprehensive investigations and carefully planned clinical trials are essential to ascertain efavirenz's function in the management of breast cancer. It will be crucial to assess efavirenz both alone and in conjunction with other proven treatments in order to determine its role in cancer practice and determine any potential benefits. Such investigations are crucial to fully understand its efficacy in treating breast cancer patients. Apart from its antitumor qualities, efavirenz has been the principal first-line antiviral treatment for over 15 years. This affords it the benefit of being a well-researched, reasonably priced therapy with a good safety record in HIV patients. If proven effective, efavirenz could offer a cost-effective therapeutic option. In conclusion, research into efavirenz as a possible treatment for breast cancer is an intriguing direction that needs additional attention.
